# Factors influencing discussion duration in breast cancer multidisciplinary team meetings: insights for streamlining care

**DOI:** 10.1007/s10549-026-08033-0

**Published:** 2026-07-20

**Authors:** Lejla Kočo, Wendelien B.G. Sanderink, Mathias Prokop, Ritse M. Mann

**Affiliations:** 1 Department of Imaging, Radboud University Medical Center, Nijmegen, Netherlands; 2 Department of Radiology, University Medical Center Groningen, Groningen, Netherlands; 3 Department of Radiology, The Netherlands Cancer Institute, Amsterdam, Netherlands

**Keywords:** Multidisciplinary team meetings (MDTMs), Breast cancer care, MDTM discussion duration, National cancer registry (NCR), Electronic medical records (EMR)

## Abstract

**Purpose:**

Multidisciplinary team meetings (MDTMs) in breast cancer care improve outcomes but are time-consuming and costly. This study investigates using data from the Dutch national cancer registry (NCR) and hospital electronic medical records (EMR) to efficiently calculate MDTM discussion durations, while complying with privacy laws.

**Methods:**

This retrospective study analyzed breast cancer MDTM discussion durations using NCR and EMR data from 2014 to 2018. Discussion times were estimated from EMR timestamps, excluding outliers, and analyzed statistically. Ethical approval was obtained, including only non-opt-out patients.

**Results:**

The final dataset included 1,048 tumors, 996 patients, and 1,487 MDTM discussion times after exclusions for missing data, duplicates, and outliers. Discussion durations varied significantly, with pre-operative and no-surgery discussions being longer than post-operative ones (*p* = 0.000), and factors such as MRI availability, tumor differentiation, malignancy, menopausal status, and cancer stage influencing duration in pre-operative cases. In post-operative discussions, molecular subtype, cancer stage, and tumor size were significant, while age, tumor differentiation, and menopausal status had no impact.

**Conclusion:**

This study evaluates discussion durations in breast cancer MDTMs using retrospective data from the NCR and EMR, demonstrating a feasible approach to assess MDTM functioning. Differences in discussion duration based on patient and tumor characteristics may help optimize MDTM efficiency.

**Clinical trial number:**

Not applicable.

## Introduction

Multidisciplinary team meetings (MDTMs) in breast cancer care are essential for ensuring evidence-based, comprehensive, combined modality treatment planning and improving patient outcomes [1–3]. Additionally, their implementation has resulted in a positive impact on the decision-making process and quality of care [4]. Existing guidelines require discussion of all new, suspect and diagnosed breast cancer cases. However, MDTMs are still time-consuming, expensive and place a heavy workload on the attending staff [5, 6]. Lack of time and high patient load have been shown to impact the quality of decision making during MDTMs, resulting in less thorough case discussions, fewer treatment options being considered, reduced multidisciplinary input, and an increased risk of suboptimal or inconsistent treatment recommendations [7, 8]. Furthermore, the gradually increasing incidence of breast cancer combined with the growing number of treatment options, and persistent shortages in healthcare staff raise concerns about the sustainability of breast cancer MDTMs [9–11]. This highlights the need to optimize both the quality and organization of breast cancer MDTMs to ensure efficiency.

The total duration of breast cancer MDTMs is determined by the discussion time allocated per patient and the total number of patients on the list. While some patients are discussed longer than others, the factors influencing these differences are not often investigated. Studies typically focus on evaluating MDTM performance and outcomes, such as decision-making quality, guideline adherence, multidisciplinary participation and patient outcomes, with discussion duration sometimes included as an indicator of time efficiency. Total meeting time or discussion duration per case is often used as an evaluation parameter to measure MDTM time efficiency or as a comparative measure. In addition, direct attendance of an observer is required in order to study how MDTMs are performed, and to collect relevant data on type of discussed cases, discussion time per case and/or meeting dynamics [12–15]. This requirement poses logistical challenges and may disrupt the natural flow of MDTMs. Within the Dutch legal and ethical framework, prospective evaluations of MDTM processes involving identifiable patient information generally require informed consent from all discussed patients, which is often not feasible in routine clinical practice. While regulations may differ between countries and depend on study design and purpose, a waiver of informed consent was not possible in this context. Retrospective analyses using routinely collected registry and medical record data therefore provide a more feasible alternative. Using coded data or anonymous observation is nearly impossible in a prospective study.

Therefore, the aim of this study is to investigate another method to collect relevant data regarding discussion duration and patient/tumor/treatment characteristics in breast cancer MDTMs. We aimed to evaluate MDTMs discussion durations and the corresponding patient characteristics, by connecting data collected from the Dutch national cancer registry (NCR) and the hospitals’ electronic medical records (EMR), in accordance with privacy laws and the law on research involving human subjects.

## Materials and methods

### Study design and study population

This study is a retrospective, single center, descriptive-analytical study. Data were retrieved from two sources and merged into a single coded database for analysis. Patients were included based on the following criteria: female, ≥ 18 years, treated for any type of breast cancer between 2014 and 2018, in an academic hospital in the Netherlands. Patients who signed an opt-out declaration (opt-out for using their data for scientific research) were automatically excluded. Patients with missing data were also excluded.

### Data sources

Patient data was obtained from the Netherlands Cancer Registry (NCR), managed by the Netherlands Comprehensive Cancer Organization (IKNL), and the hospital’s electronic medical record (EMR) system EPIC (Epic Systems Corporation, Verona, WI, USA). Different sets of variables were collected from both sources for the same population. The NCR registers all newly diagnosed cancer cases in the Netherlands and provides customized research data in compliance with Dutch privacy laws offering extensive clinical data for research purposes [16]. A pseudonymized research dataset was requested containing a total of 126 variables from the NCR [17]. Data from the hospital’s EMR system were collected within a digital research environment using CliniQuest, a clinical data collection platform that uses queries to extract the necessary information directly from the EMR without reporting privacy sensitive information.

### Data collection and calculation of discussion duration

The NCR provided patient variables including patient, tumor, diagnostic and treatment characteristics (see Supplementary table S1 for a complete list of variables). The data to calculate discussion duration was extracted from the hospitals EMR, this included all dates, timestamps of the ‘report saving instant’ during the breast cancer MDTMs. Only the data of the patients that were also in the NCR database were identified, all other timestamps were labeled as ‘NULL’. No patient data whatsoever could be traced from these timestamps. The raw timestamp database was cleaned in order to only get relevant timestamps, i.e. only the dates and times that fall within the weekly MDTM timeframe.

To calculate the discussion duration, we used the assumption that the time between two subsequent saving instants of the digital MDTM report of two different patients would sufficiently estimate the duration of each discussion (Table 1). Since there is no previous saving instant for the first discussion, there is no way to calculate discussion duration for the first patient in the MDTM, therefore all first discussions were excluded. NULL timestamps were required to account for missing patients still discussed during an MDTM. These patients either opted-out or were not included in the NCR patient population.

**Table 1:** Example of the discussion duration calculation process. The content of this table is fictitious and serves as an example. Discussion duration was calculated by subtracting the previous timestamp (row #3 - #2), this was repeated for the whole MDTM. First discussion were invalid since there is no previous timestamp to determine discussion duration. Green rows represent timestamps that would be coupled to patient data from the NCR and used in the analysis

#	Pseudonym ID	Date	Time ‘report saving instant’	Calculated discussion duration
1	630,546,392	6-2-2015	14:59:52	N/A
2	NULL	6-2-2015	15:02:08	00:00:36
3	574,723,847	6-2-2015	15:04:21	00:02:13
4	NULL	6-2-2015	15:07:36	00:03:15
5	324,823,846	6-2-2015	15:11:07	00:03:31

Discussion durations outside the central 95% of the distribution (i.e., below the 2.5th percentile and above the 97.5th percentile) were considered potential recording errors and excluded from the analysis. For instance, a calculated discussion duration of 3 s is most likely a record that was not discussed but opened by accident. The rows with NULL pseudonyms and missing patient information were also excluded. Furthermore, all timestamps outside the scheduled MDTM window were excluded, since some reports are edited by the notetaker or the treating physician after the meeting is finished.

### Data analysis

Data analysis was performed using SPSS software (IBM SPSS statistics, version 28). Discussion duration unit was changed from hh: mm: ss to seconds for further analysis. An overview of variables used in the data analysis is shown in Table 2.

**Table 2:** Overview of variable types used for data analysis

Discrete variables	Categorical variables (2 categories)	Categorical variables (> 2 categories)
• Discussion time in seconds (dependent variable) • Age in years • Tumor size in mm	• Pre- or post-operative MDTM discussion • Post-MRI MDTM discussion (yes/no) • DCIS component (yes/no) • Tumor behavior (malignancy/carcinoma in situ)	• Menopausal status • BIRADS score • Morphology tumor • Differentiation tumor • Stage • Molecular subtype • Discussion pre- post- or during neoadjuvant chemotherapy

Continuous data variables were checked for distribution (histogram and Kolmogorov-Smirnov test). None of the discrete variables were normally distributed: discussion duration (D(1499) = 0.116, *p* < 0.001), age (D(1487) = 0.055, *p* < 0.001) and tumor size (D(1297) = 0.151, *p* < 0.001). Since the variables of discussion time, age, and tumor size were not normally distributed, Spearman’s rank-order correlation was used to analyze numeric values and scale variables individually.

The effect of individual patient and tumor characteristic on discussion duration was determined using the Mann-Whitney U-test for categorical variables with two categories and the Kruskal-Wallis test for categorical variables with more than two categories.

### Ethical considerations and data management

Ethical approval to perform this research was obtained from the local medical ethical committee (CMO 2019–5371). The key file containing patient IDs and study pseudonyms is stored on a separate location and was exclusively accessible to the study team.

## Results

### Database demographics

**Fig. 1:**
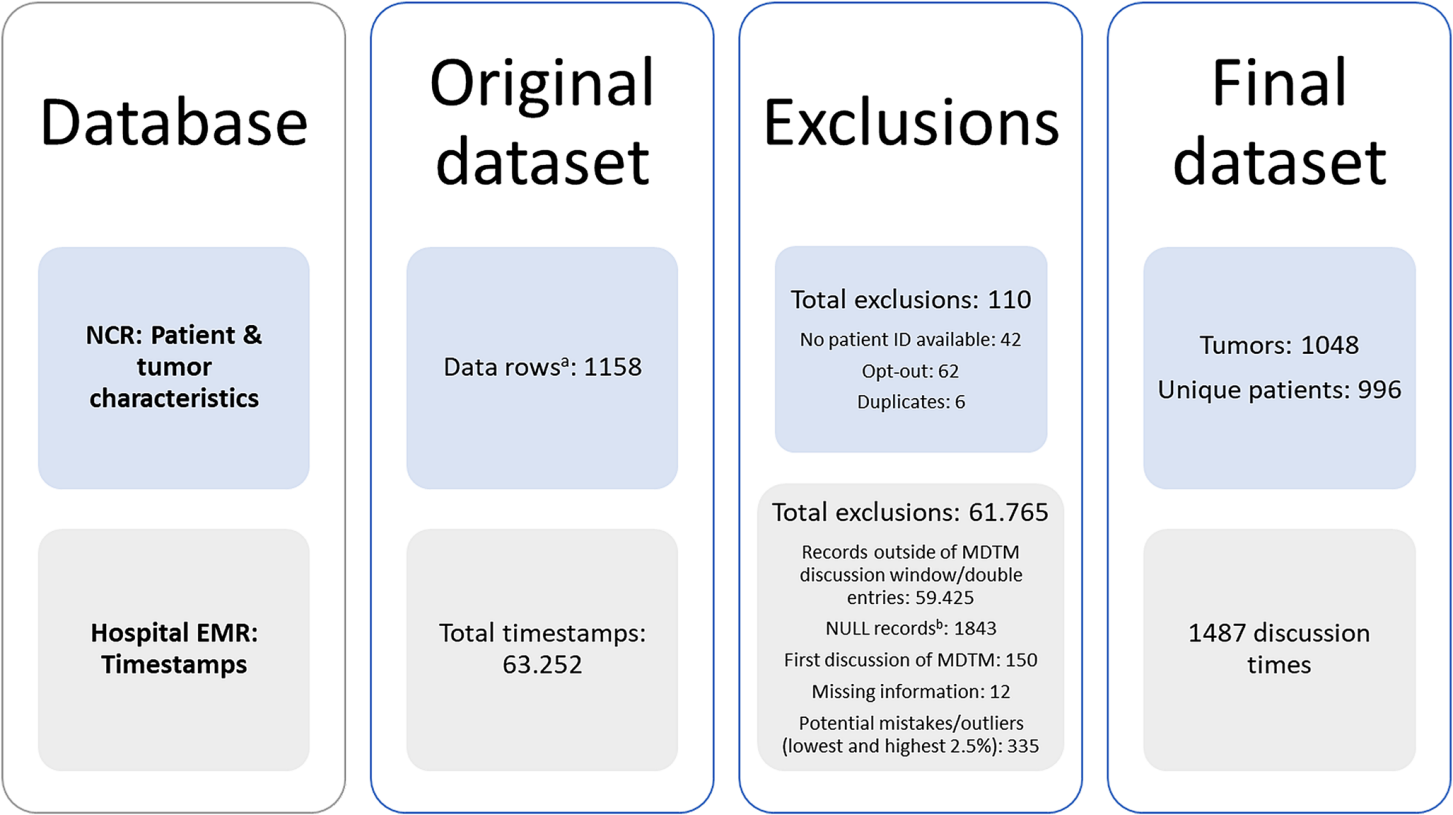
Flowdiagram of dataset cleaning.^a^: One data row corresponds with one registered tumor, a patient can have multiple tumors. ^b^: NULL records are timestamps that were not coupled to a patient ID, refer to the materials and methods section for more information. Outliers were defined as discussion durations below the 2.5th percentile and above the 97.5th percentile

The NCR provided a list of 1158 tumors treated in the evaluated period. Exclusions on patient-level were carried out due to missing IDs, opt-outs and duplicates. Timestamp exclusions involved removing records outside of the MDTM discussion window, duplicates, records without patient ID, first discussions, missing information and probable outliers. The final dataset comprised 996 patients, 1,048 tumors and 1,487 discussion times (Fig. 1).

The mean age of the patients was 57 years (ranging: 26–96 years). The duration of the discussions ranged from 00:38 to 11:00 (mm: ss), with a mean duration of 03:35 and a median of 03:01. The total population was divided into three subgroups based on the distinct goals of the discussions: pre-operative discussions, post-operative discussions and discussions about patients that did not undergo surgical treatment (referred to as the ‘No surgery group’). The pre-operative group included 727 discussions, the post-operative group comprised 648 discussions and the no surgery group consisted of 112 discussions. Within the pre-operative group, the mean discussion time was 03:58 (median: 03:22), while post-operative discussions had a mean of 03:01 (median: 02:27). Discussions in the no-surgery group were the longest, with a mean duration of 04:16 (median: 03:50). Characteristics of the total population and the respective subgroups can be found in Table 3.

**Table 3:** Overview of database content. The table depicts the N of discussion times per variable category and the mean discussion duration within that subgroup in brackets (m: ss), unless otherwise specified. *Tumor size data was reported in the NCR for only one patient. Superscripts indicate subgroup-specific statistical comparisons. ^a^: p‑value for comparisons within the pre‑operative MDTM group. ^b^: p‑value for comparisons within the post‑operative MDTM group. For binary variables (e.g. MRI availability), differences in discussion duration were tested within pre-operative and post-operative groups separately, not between these groups

	Total population	Pre-operative group	Post-operative group	No surgery group	*P*-value (within subgroup)
MDTM discussions and duration					
Total discussions	1,487	727	648	112	^*^ *P*=0.000
Number of MDTMs	217	201	203	88	
Number of patients	647	478	487	63	
Mean discussion duration	03:35	03:58^*^	03:01^*^	04:16^*^	
Median discussion duration	03:01	03:22	02:27	03:50	
Range discussion duration	00:38–11:00	00:38–11:00	00:38–10:27	00:51–10:35	
Age					
Mean	57 years	55 years	57 years	66 years	^a^ r(727) = -0.137, *p* < 0.001 ^b^ r(648) = -0.028, *p* = 0.473
Median	55 years	54 years	56 years	64 years	
Range:	26–96 years	26–96 years	26–96 years	31–93 years	
< 40:	142 (4:00)	79 (4:41)^a^	56 (2:57)^b^	7 (4:46)	
40–49:	314 (3:48)	170 (4:12)^a^	127 (3:17)^b^	17 (3:40)	
50–59:	430 (3:33)	215 (4:00)^a^	196 (3:00)^b^	19 (4:08)	
60–69:	351 (3:23)	169 (3:36)^a^	164 (2:57)^b^	18 (5:07)	
$\:\ge\:$70:	250 (3:27)	94 (3:40)^a^	105 (3:02)^b^	51 (4:09)	
Menopausal status					
Pre-menopausal	318 (4:03)	186 (4:40)^a^	121 (3:01)^b^	11 (4:54)	^a^ *p* < 0.001 ^b^ *p* = 0.628
Menopausal	46 (3:39)	20 (4:02)	26 (3:22)^b^	0	
Post-menopausal	734 (3:34)	335 (3:47)^a^	325 (3:09)^b^	74 (4:25)	
Unknown	389	186	176	27	
Tumor size					
Mean	22 mm	21 mm	23 mm	34 mm	^a^ r(688) = 0.018, *p* = 0.629 ^b^ r(608) = 0.097, *p* = 0.017
Median	16 mm	16 mm	17 mm	34 mm	
Range	0–131 mm	0–120 mm	0–131 mm	34 mm *	
0–8	296 (3:34)	170 (4:05)^a^	126 (2:54)^b^	-	
9–15	324 (3:16)	165 (3:42)^a^	159 (2:48)^b^	-	
16–27	346 (3:36)	187 (4:02)^a^	159 (3:05)^b^	-	
28–131	331 (3:37)	166 (4:00)^a^	164 (3:14)^b^	-	
Unknown	190	39	40	111	
BIRADS score					
0	4 (5:43)	2 (5:33)^a^	2 (5:53)^b^	0	^a^ *p* = 0.309 ^b^ *p* = 0.015
1	11 (3:35)	5 (3:36)^a^	5 (3:59)^b^	1 (1:30)	
2	8 (3:32)	7 (3:25)^a^	0	1 (4:22)	
3	51 (3:42)	26 (4:25)^a^	25 (2:58)^b^	0	
4	620 (3:23)	307 (3:48)^a^	272 (2:49)^b^	41 (4:09)	
5	763 (3:43)	373 (4:06)^a^	324 (3:08)^b^	109 (4:20)	
Unknown	30	7	20	3	
Tumor type					
Carcinoma in situ	190 (2:47)	78 (3:08)^a^	87 (2:15)^b^	25 (3:32)	^a^ *p* = 0.952 ^b^ *p* = 0.001
Invasive breast cancer	1297 (3:42)	649 (4:05)^a^	561 (3:09)^b^	87 (4:29)	
DCIS component					
Yes	838 (3:41)	420 (4:04)^a^	385 (3:09)^b^	33 (4:57)	^a^ *p* = 0.759 ^b^ *p* = 0.574
No	450 (3:45)	226 (4:07)^a^	170 (3:09)^b^	54 (4:11)	
Unknown	199	81	93	25	
Morphology					
Ductal carcinoma	1019 (3:35)	497 (4:00)^a^	447 (2:56)^b^	75 (4:40)	^a^ *p* = 0.952 ^b^ *p* = 0.100
Lobular carcinoma	241 (3:29)	116 (3:57)^a^	97 (2:56)^b^	28 (3:26)	
Ductal and lobular carcinoma	116 (3:45)	65 (3:50)^a^	51 (3:38)^b^	0	
Other	111 (3:40)	49 (4:02)	53 (3:02)	9 (3:33)	
Differentiation status					
High	179 (3:12)	87 (3:26)^a^	89 (2:58)^b^	3 (3:29)	^a^ *p* = 0.002 ^b^ *p* = 0.227
Moderate	497 (3:14)	241 (3:40)^a^	249 (2:49)^b^	7 (2:59)	
Poor	428 (3:44)	217 (4:14)^a^	200 (3:08)^b^	11 (4:48)	
Unknown	383	182	110	91	
Stage					
Stage 0	190 (2:47)	78 (3:08)^a^	87 (2:15)^b^	25 (3:32)	^a^ *p* < 0.001 ^b^ *p* < 0.001
Stage I	500 (3:20)	247 (3:44)^a^	240 (2:49)^b^	13 (5:12)	
Stage II	526 (3:39)	288 (4:01)^a^	215 (3:06)^b^	23 (3:59)	
Stage III	192 (4:31)	98 (4:53)^a^	86 (4:09)^b^	8 (4:04)	
Stage IV	69 (4:39)	13 (5:34)^a^	14 (3:44)^b^	42 (4:40)	
Unknown	10	3	6	1	
Molecular subtype					
Luminal A	831 (4:12)	417 (3:55)^a^	369 (3:00)	45 (3:54)	^a^ *p* = 0.085 ^b^ *p* = 0.043
Luminal B	222 (2:15)	96 (4:19)^a^	89 (3:29)	37 (5:13)	
Her2-positive	57 (1:37)	37 (4:23)^a^	19 (3:24)	1 (1:30)	
Triple negative	158 (5:36)	84 (4:26)^a^	70 (3:29)	4 (4:52)	
Unknown	219	93	101	25	
MRI available prior to MDTM					
Yes	1312 (3:30)	568 (3:57)^a^	647 (3:01)^b^	97 (4:12)	^a^ *p* = 0.049 ^b^ *p* = 0.142
No	175 (4:11)	159 (4:07)^a^	1 (6:33)^b^	15 (4:40)	

### Discussion durations

We found that pre-operative and no-surgery discussions were significantly longer than post-operative discussions (*p* = 0.000). Additional analyses were conducted within the pre-operative and post-operative subgroups using the variables and statistical tests listed in the Materials and Methods section. Due to a combination of smaller sample sizes and missing data in the No surgery group, no comparisons were made within that subgroup.

### Pre-operative group

Discussions for patients before surgical treatment tended to last significantly longer in several situations. Cases with carcinoma in situ (CIS) were linked to significantly shorter discussions lasting 03:08, compared to cases with invasive breast cancer (04:05, *p* = 0.001). Similarly, patients with poorly differentiated tumors needed longer discussions, averaging 04:14, compared to those with well differentiated (3:26) and moderately differentiated tumors (03:40) (*p* = 0.002). Pre-menopausal patients needed longer discussions too, averaging 04:40, compared to 03:47 for post-menopausal patients (*p* < 0.001). Cancer stage also played a role, with discussions for stage 4 patients lasting around 05:34, significantly longer than the 03:08 for stage 1 patients (*p* < 0.001). When an MRI scan had not yet been performed, discussions lasted about 04:07, while they were shorter (around 03:57) when MRI imaging was already available (*p* = 0.049). There was a notable trend where older age was linked to shorter discussions (r(727) = -0.137, *p* < 0.001).

Some factors had no significant impact on discussion length, including molecular subtype (*p* = 0.085), the presence of a DCIS component (*p* = 0.759), tumor type (*p* = 0.952) BIRADS score (*p* = 0.309), tumor morphology (*p* = 0.952) and tumor size (r(688) = 0.018, *p* = 0.629).

### Post-operative group

Discussions for patients after their surgery showed significant variation for molecular subtype (*p* = 0.043) BIRADS score (*p* = 0.015) and cancer stage (*p* < 0.001). Again, patients with invasive breast cancer had much longer discussions, averaging 03:09, compared to those with carcinoma in situ, for whom discussions lasted around 02:15 (*p* < 0.001). Furthermore, there was a small but statistically significant positive correlation between tumor size and discussion duration, meaning that as tumor size increased, so did the length of conversations (r(608) = 0.097, *p* = 0.017).

Likewise, certain factors had no significant impact on how long discussions lasted. Age, for example, did not correlate with discussion duration (r(648) = -0.028, *p* = 0.473), nor did tumor differentiation grade (*p* = 0.227), the presence of a DCIS component for invasive cancers (*p* = 0.574), tumor type (*p* = 0.100), tumor morphology (*p* = 0.100) or menopausal status (*p* = 0.628).

## Discussion

### Key findings

The main goal of this study was to explore the discussion duration per patient during multidisciplinary team meetings (MDTMs) using available data, and without disrupting the normal flow of the meetings. Discussions regarding pre-operative patients and those not undergoing surgery were significantly longer than those for post-operative patients. Within the pre-operative group, longer discussions were associated with invasive vs. non-invasive breast cancer, poorly differentiated tumors, advanced cancer stages, pre-menopausal patients, and the absence of MRI images. Older age correlated with shorter discussions. In the post-operative group, longer discussions were linked to molecular subtype, malignancy, and tumor size.

### Findings in relation to clinical practice

The findings in this study provide an overview of the functioning of MDTMs in clinical practice and are consistent with patterns commonly observed during MDTM discussions. Pre-operative and no-surgery discussions involve more complex decision-making than post-operative discussions. In the pre-operative phase, treatment plans are still being developed, requiring detailed discussions to evaluate diagnostic information, treatment options, and risks. This could explain why missing MRI images, poorly differentiated tumors, and advanced cancer stages result in longer discussions—clinicians need more time to assess incomplete or complex cases.

Discussions for pre-menopausal patients may take longer due to considerations like fertility preservation or hormone-sensitive therapies. In contrast, shorter discussions with older patients may reflect more straightforward treatments or established protocols, leading to quicker decisions.

Post-operative discussions are generally shorter, as the treatment path is clearer, focusing on surgery outcomes and follow-up. However, complex characteristics such as specific molecular subtypes or larger tumors still require longer discussions due to potential impacts on follow-up treatments like chemotherapy or radiation, but also because of increasingly individualized post-operative decision-making involving endocrine and targeted therapies, genomic testing, germline mutation status, and deliberations on the intensity and duration of adjuvant treatment.

The lack of significant effects from variables like age or tumor differentiation in post-operative cases suggests treatment decisions are more standardized at this stage or were already pre-discussed in the pre-operative stage. The nature of post-surgical discussions therefore appears to be somewhat more routine when compared to the complexity of pre-surgery decisions.

### Potential applications of retrospective calculation of discussion durations

Understanding average discussion durations for different types of cases may provide valuable insights for optimizing MDTM planning. First, estimating the time required for specific discussions enables more precise scheduling, ensuring that complex cases receive sufficient attention without causing the meeting to overrun. This approach could help balance the time allocated to all cases, maintaining high-quality discussions across the board [18]. By anticipating complex cases, the agenda could be adjusted accordingly, potentially reducing decision fatigue by distributing these discussions throughout the meeting. Increased awareness of estimated discussion times may also be relevant in the context of MDTM preparation, as qualitative research has shown that incomplete case information and insufficient preparation can lead to delays during MDTMs and necessitate postponement of discussions [19].

Studies have explored various other strategies to improve or streamline MDTMs. One approach is to focus specialist time on complex cancer cases and discuss less critical cases briefly or not at all. However, many participants oppose this, citing concerns that reduced discussion could compromise care quality and safety, especially when individual clinicians, rather than teams, make treatment decisions [20, 21]. Our study also does not provide handholds for non-discussion of subsets of patients as even for cases that appear relatively simple according to our analysis, such as smaller cancers in older patients, or patients with DCIS only, average discussion times are not negligible. In this context, our findings place these proposed strategies in perspective, as the observed variability in discussion duration confirms that case complexity is reflected in MDTM discussions, while also demonstrating substantial overlap in discussion time across patient subgroups.

Another suggestion is to group cases so specialists attend only the relevant portions of the MDTM or hold separate MDTMs for different case types [20]. This is somewhat supported by our study, particularly separating pre-operative and post-operative discussions may enhance the flow of the meeting. Other studies reported that reducing the number of cases discussed did not lead to higher-quality discussions, indicating this may not be an optimal solution [18], however, our analysis suggests that, within the studied setting, no more than 20–25 patients can be discussed per hour. Support for streamlining also varies by cancer type, with urology MDTs being more supportive to streamlining compared to lung, breast, or colorectal specialists. This may be due to clearer protocols, less complex cases, or larger caseloads in urology driving the need for change [21].

### Study limitations

Our study is a single center analysis. While the number of MDTM discussion evaluated is large, and obtained over a long time, thus providing a reliable average of discussion times per tumor, variations exist in how these meetings are conducted across different hospitals [10, 11, 19]. Factors such as team composition, hospital size, care processes, and patient population can influence the workflow. The impact of these variations on MDTM outcomes is still poorly understood, indicating that developing a universal strategy for improvement of efficiency based on our data alone may not be appropriate [22, 23].

A further notable limitation of this study is the assumption we had to make regarding the exact duration and content of discussions. While the data provides a general reflection of clinical practice, we were unable to verify the exact topics discussed during MDTMs or the accuracy of the recorded durations. Patients may be discussed only partially and postponed to a later meeting, and the MDTM may issue multiple recommendations based on different clinical scenarios, with the final choice dependent on the availability of additional information at a later stage. Moreover, it is not possible to assess the quality of the discussions retrospectively as one would by using observational methods [24, 25]. These assumptions were necessary due to the nature of our dataset and privacy constraints.

### Future research

The ability to calculate MDTM discussion durations using existing data opens up new possibilities for retrospective studies and MDTM evaluations. This is particularly valuable in cases where real-time observation may not be possible or where compliance with privacy laws must be strictly followed. The availability of this data also facilitates more efficient research, potentially enabling studies that cover a broader scope and involve larger datasets without additional strain on clinical resources.

This study shows that calculating MDTM discussion durations from NCR and EMR data is a promising method for research purposes, particularly in contexts where privacy concerns are essential. Future research should focus on further refining these calculation methods and exploring their broader applicability. Additionally, the insights gained from this approach could lead to the development of new analytical tools for studying MDTMs in more detail. Potentially, it is possible to automate the generation of such reports for any hospital, thus providing practical benchmarks for local organization and optimization of MDTMs.

## Conclusion

This study evaluates discussion durations in breast cancer MDTMS and reflects clinical practice. This shows that using retrospective, existing data from sources such as the national cancer registries (NCR) and electronic medical records (EMR) can be a feasible approach for gathering insights in MDTM functioning. Highlighted differences in discussion duration based on patient and tumor characteristics may be used to optimize the functioning of MDTMs. 

## Data Availability

The data that support the findings of this study were derived from the Netherlands Cancer Registry (NCR), managed by the Netherlands Comprehensive Cancer Organisation (IKNL). Restrictions apply to the availability of these data, which were used under license for the current study and are not publicly available. Data may be available from IKNL upon reasonable request and with permission of IKNL.
